# Rapid Low-Cost Microarray-Based Genotyping for Genetic Screening in Primary Immunodeficiency

**DOI:** 10.3389/fimmu.2020.00614

**Published:** 2020-04-15

**Authors:** Narissara Suratannon, Rogier T. A. van Wijck, Linda Broer, Laixi Xue, Joyce B. J. van Meurs, Barbara H. Barendregt, Mirjam van der Burg, Willem A. Dik, Pantipa Chatchatee, Anton W. Langerak, Sigrid M. A. Swagemakers, Jacqueline A. C. Goos, Irene M. J. Mathijssen, Virgil A. S. H. Dalm, Kanya Suphapeetiporn, Kim C. Heezen, Jose Drabwell, André G. Uitterlinden, Peter J. van der Spek, P. Martin van Hagen, The South East Asia Primary Immunodeficiencies (SEAPID) Consortium

**Affiliations:** ^1^Department of Immunology, Laboratory Medical Immunology, Erasmus MC, University Medical Center, Rotterdam, Netherlands; ^2^Pediatric Allergy & Clinical Immunology Research Unit, Division of Allergy and Immunology, Department of Pediatrics, Faculty of Medicine, Chulalongkorn University, King Chulalongkorn Memorial Hospital, The Thai Red Cross Society, Bangkok, Thailand; ^3^Department Internal Medicine, Division of Clinical Immunology, Erasmus MC, University Medical Center, Rotterdam, Netherlands; ^4^Genetic Laboratory and Human Genomics Facility HuGeF, Department of Internal Medicine, Erasmus MC, University Medical Center, Rotterdam, Netherlands; ^5^Academic Center for Rare Immunological Diseases (Rare Immunological Disease Center, RIDC), Erasmus MC, University Medical Center, Rotterdam, Netherlands; ^6^Laboratory for Immunology, Department of Pediatrics, Leiden University Medical Centre, Leiden, Netherlands; ^7^Department of Pathology & Clinical Bioinformatics, Erasmus MC, University Medical Center, Rotterdam, Netherlands; ^8^Department of Plastic and Reconstructive Surgery, Erasmus MC, University Medical Center, Rotterdam, Netherlands; ^9^Center of Excellence for Medical Genomics, Division of Medical Genetics and Metabolism, Department of Pediatrics, Faculty of Medicine, Chulalongkorn University, Bangkok, Thailand; ^10^Excellence Center for Genomics and Precision Medicine, King Chulalongkorn Memorial Hospital, The Thai Red Cross Society, Bangkok, Thailand; ^11^International Patient Organization for Primary Immunodeficiencies (IPOPI), Downderry, United Kingdom

**Keywords:** primary immunodeficiencies, microarray-based genotyping, SNP microarray, single nucleotide variants (SNV) calling, copy number variants (CNV) calling

## Abstract

**Background:** Genetic tests for primary immunodeficiency disorders (PIDs) are expensive, time-consuming, and not easily accessible in developing countries. Therefore, we studied the feasibility of a customized single nucleotide variant (SNV) microarray that we developed to detect disease-causing variants and copy number variation (CNV) in patients with PIDs for only 40 Euros.

**Methods:** Probes were custom-designed to genotype 9,415 variants of 277 PID-related genes, and were added to the genome-wide Illumina Global Screening Array (GSA). Data analysis of GSA was performed using Illumina GenomeStudio 2.0, Biodiscovery Nexus 10.0, and R-3.4.4 software. Validation of genotype calling was performed by comparing the GSA with whole-genome sequencing (WGS) data of 56 non-PID controls. DNA samples of 95 clinically diagnosed PID patients, of which 60 patients (63%) had a genetically established diagnosis (by Next-Generation Sequencing (NGS) PID panels or Sanger sequencing), were analyzed to test the performance of the GSA. The additional SNVs detected by GSA were validated by Sanger sequencing.

**Results:** Genotype calling of the customized array had an accuracy rate of 99.7%. The sensitivity for detecting rare PID variants was high (87%). The single sample replication in two runs was high (94.9%). The customized GSA was able to generate a genetic diagnosis in 37 out of 95 patients (39%). These 37 patients included 29 patients in whom the genetic variants were confirmed by conventional methods (26 patients by SNV and 3 by CNV analysis), while in 8 patients a new genetic diagnosis was established (6 patients by SNV and 2 patients suspected for leukemia by CNV analysis). Twenty-eight patients could not be detected due to the limited coverage of the custom probes. However, the diagnostic yield can potentially be increased when newly updated variants are added.

**Conclusion:** Our robust customized GSA seems to be a promising first-line rapid screening tool for PIDs at an affordable price, which opens opportunities for low-cost genetic testing in developing countries. The technique is scalable, allows numerous new genetic variants to be added, and offers the potential for genetic testing not only in PIDs, but also in many other genetic diseases.

## Introduction

Primary immunodeficiency disorders (PIDs) are a heterogeneous group of diseases, including more than 400 distinct monogenic inherited disorders, that affect the development and function of the immune system (1–3). Obtaining a genetic diagnosis in PID patients is crucial for providing an optimal standard of care and personalized treatment tailored to specific molecular defects (4). Current genetic diagnostic approaches for PIDs are based on Sanger sequencing, next-generation sequencing (NGS), and copy number variant (CNV) analysis. However, these techniques are time-consuming, costly and involve complicated data interpretation. Due to high costs and resource limitations, not all genetic tests are available in developing countries; therefore, rapid, robust, and inexpensive molecular tools must be developed to meet this need.

Single nucleotide polymorphism (SNP) arrays are high-throughput DNA microarrays that originated from the early 2000s and are a powerful platform for simultaneously analyzing hundreds of thousands of SNPs and evaluating CNVs in a single experiment (5). Recently, the cost of SNP arrays has decreased substantially (from 300 Euro to 40 Euros per sample), driven by the very large sample sizes needed to perform genome-wide association studies (GWAS). Both Affymetrix/Thermofisher and Illumina have designed cost-effective arrays that contain ~800,000 variants, allowing a wide range of genetic variants to be assessed.

To our knowledge, this “proof of principle study” is the first to use the Illumina Global Screening Array (GSA; v1) to detect rare Mendelian mutations, consisting of single nucleotide variants (SNVs), small insertions and deletions (INDELs) and CNVs, rather than SNPs. To the multi-ethnic genome-wide GSA v1, we added 9,415 custom variants within the 277 validated PID genes listed in the International Union of Immunological Studies (IUIS) 2015 (6) to capture pathogenic variants. We then analyzed 151 blood DNA samples derived from 95 patients clinically diagnosed with PIDs and 56 non-PID controls that had previously undergone whole-genome sequencing (WGS) at 80x coverage, enabling the GSA results to be technically validated by comparison with the WGS data.

## Materials and Methods

### Array Design

The Illumina Custom GSA was produced as a research use-only tool in San Diego, USA, ~50,000 positions for a custom design. Our array contained custom content representing all variants and INDELs at the time of manufacture in the 277 PID-related genes described by IUIS 2015 (6), derived from the licensed human gene mutation database (HGMD) professional (7). Of the 10,250 variants selected, 9,415 (91.9%) were successfully placed on the array by Illumina. The aim of this custom part of the array was to identify the known PID variants by calling SNVs and CNVs in these PID genes. In addition, the Illumina GSA contains a multi-ethnic genome-wide backbone of SNPs with 696,375 probes, which enables genome-wide CNV calling.

### Sample Selection and DNA Preparation

On the first run, a total of 95 patients clinically diagnosed with PIDs from the Erasmus Medical Center PID Biobank/Clinical Repository were randomly selected to test the performance of the array to diagnose PIDs. A detailed description of the PID patients is shown in Supplementary (Tables S1, S2). All patients had undergone conventional genetic testing by either targeted NGS PID panel or Sanger sequencing based on their clinical phenotype. Seventy-one SNVs and/or small INDELs and four exon deletions including 3 homozygous exon deletions and 1 hemizygous deletion were discovered. A genetic diagnosis was established in 60 of the 95 patients (63%). The remaining 35 patients were unresolved, indicating the possibility of an uncharacterized genetic variant. In 7 of these 35 patients, some variants were discovered (patient no. 1–7, Table S2); however, these were not found to be sufficient enough to account for the clinical phenotype based on the inheritance pattern of the disease. A single sample of a healthy individual that had undergone WGS sequencing was added on the first run for array validation (HapMap NA 19240). DNA was extracted from blood using a DNA Isolation Kit (Qiagen, Valencia, CA, USA). On the second run of the array, DNA samples of 41 PID patients (that were also included in the first run) and 55 non-PID controls which had pre-existing WGS data were selected. All samples were registered by the biobanking and biomolecular resources research infrastructure (BBMRI) and/or consent for molecular diagnostic testing according to the Helsinki Guidelines.

### Validation of Array-Based Genotyping

For validation of the genotype calling of the customized array, we used one HapMap sample, which has been investigated to benchmark various WGS platforms at the Erasmus University Medical Center (8) and 55 samples with pre-existing WGS data reaching an average depth of 80x. These samples were originated from unrelated parents of a genetic study of craniofacial malformations (9). For these 56 non-PID samples that previously had undergone WGS, SNV position genotypes of the custom probes were called from the BAM files, using SAMtools/ BCFtools (Li, 2011), and were compared to the genotype called from the array for the same sample. Probes with different callings between the array and the WGS for these 56 control samples were excluded from analysis because of the possibility of probe malfunction.

### Genetic Data Analysis

#### Array Quality Control

Two IDAT files (green, red) were generated per sample and uploaded on to Illumina GenomeStudio 2.0 software prior to quality control (QC) analysis using PLINK (v1.9) (10). QC analysis tested for genotyping efficiency per SNP at a threshold of 97.5%, with the sample call rate set at 97.5% and deviations from Hardy-Weinberg equilibrium defined as *P* < 1 × 10^−4^. Additionally, zCall was performed to improve rare variant calling (11), after which a stringent SNP and sample call rate filter of 98% was applied. Ethnic origin and sex/gender were used to control for data integrity during sample processing and analysis.

#### SNV Calling

Detailed variant analysis for SNV detection was performed on post-QC data using PLINK (v1.9), GenomeStudio 2.0, and R-3.4.4. Only disease-causing variants according to HGMD classifications were selected. Next, variants were filtered against the gnomAD database with a frequency threshold of below 0.5 %. Variants that occurred more than once in the dataset were excluded as controls for the effect of faulty probes, while all X-linked heterozygous calls in males were excluded, these would lead likely to false-positive results. Lastly, all remaining variants were manually checked using genotyping module SNP graphs generated by GenomeStudio 2.0 to determine whether the genotype call matched the expected genotype by their signal intensity, if not the variant was excluded.

#### CNV Calling

CNV analysis was carried out using the SNP-FASST2 Segmentation Algorithm within BioDiscovery Nexus Copy Number Discovery Version 10.0 (El Segundo, USA). Large chromosomal aberrations were visually identified by making log-ratio and B-allele frequency plots in order to detect deletions, amplifications, polyploidy or long-contiguous stretch of homozygosity (LCSH). For small CNVs, the significance threshold for CNV calling within Nexus 10.0 was set at 5 ×10^−6^ requiring a minimum of 4 probes per segment and a maximum contiguous probe spacing of 1,000 Kbp. Log_2_ ratio values of −0.3 and −1.1 were used to detect single and more than two copy losses, respectively, while values of 0.2 and 0.7 were used to detect single and more than two copy gains. Only CNVs, that were located in the 277 PID according to the IUIS classification genes were selected. Next, these CNVs were filtered against the Nexus database for known CNVs. For heterozygous CNVs, the overlap with known CNVs was set to 0%, whereas no threshold was used for homozygous CNVs. Lastly, a frequency filter was applied such that only CNVs occurring once in the dataset were selected to filter any CNVs that arose due to probe malfunction when capturing CNVs.

#### Validation by Sanger Sequencing and Calculation of Diagnostic Yields

Sanger sequencing was performed to validate all newly detected SNVs. The sensitivity (of previously known variants) and the overall sensitivity (including the addition of newly detected “Sanger confirmed” variants) was defined as the proportion of SNVs and CNVs that could be replicated in both techniques. The diagnostic yield was calculated at the level of patients when a genetic diagnosis could be made. Reaching a conclusive genetic diagnosis is based on identifying a genetic variant with an inheritance pattern that matches the inheritance pattern reported in the Online Mendelian Inheritance in Man (OMIM) database, on observing close correlation with the clinical PID phenotypes, and/or on mentioning of the variant in the IUIS database.

#### Assessment of the Reproducibility

To assess the reproducibility of the array, we focused only on the custom content of the GSA and compared the post-QC data for the 41 overlapping PID samples in the two array runs. The genotypes acquired in the two runs were compared for the same sample.

## Results

### Overall Technical Array Performance

We performed two array genotyping experiments, where DNA samples were on the array (run 1 and run 2) to allow inter-assay comparison. Of the 9,415 custom variants added to the standard GSA v1 content, 8,883 and 8,852 variants were included in the post-QC data of the custom content for the first and second runs, respectively. In the second run, 3 samples (2 PID and 1 non-PID) were excluded from further analysis as they failed to achieve the sample call rate threshold of 98%, leaving 39 PID samples for inter-assay validation and, 55 samples from non-PID healthy controls (in whom WGS was performed) for comparison of the array genotype calling with WGS genotype callings. The correlation between the genotype of the custom SNVs in the PID array and WGS data of the 55 non-PIDs was robust. Of the 8,852 post-QC variants in the second run, 1,902 probes captured small (INDELs) and 6,950 probes captured SNVs. These 6,950 variants were subsequentially checked against the WGS data (of all non-PID controls) and genotyping of 6,928 (99.7%) of the variants on the custom GSA v1 matched that acquired by WGS (at 80x coverage). Subsequently, we checked all the non-reference genotype SNV calls made by the GSA (*n* = 2,626) as these would be identified as positive results and observed that only 11 (0.15%) did not match the genotype called by WGS. Moreover, these 2,626 calls were almost exclusively reported as benign variants/polymorphisms in HGMD.

### SNV Analysis in PID Patients

We then analyzed the array-based genetic diagnosis in 95 patients with clinically diagnosed PIDs. Our customized GSA v1 detected 80 SNVs or small INDELs, of which 30 variants were originally detected by conventional methods (**Figure 2** and Table S3). The diagnosis was confirmed in 26 out of 60 patients who had a previously established genetic diagnosis (Table S1). In 31 patients, the array could not replicate the genetic variants found by conventional diagnostics. Seven of these 31 patients had variants that could have been measured by the specific probes on the array, but the GSA failed to do so, because these probes did not pass the stringent QC after rare-variant calling performed with zCall. For 2 of these 31 patients, the GSA was only able to identify 1 of 2 variants as these patients were compound heterozygous. For the remaining 21 patients (29 variants as some patients had multiple variants), the GSA could not replicate the SNV and/or INDEL as the variants were not known at the time of GSA manufacturing process and therefore no probes were added to the array to investigate these variants.

Interestingly, in 35 patients whom conventional diagnostics did not find a causative genetic variant (Table S2), our customized GSA could detect pathogenic variants and lead to a diagnosis in 6 patients. These variants included heterozygous variants in *signal transducer and activator of transcription 1 (STAT1), colony stimulation factor 3 receptor (CSF3R), tumor necrosis factor receptor superfamily member 13B (TNFRSF13B), inhibitor of nuclear factor-kappa B kinase subunit gamma (IKBKG), C-X-C chemokine receptor type 4 (CXCR-4)*, and a homozygous mutation in *vacuolar protein sorting 45 homolog (VPS45)*. These genes were not analyzed as the potential genetic cause during conventional diagnostics, possibly due to overlapping clinical phenotypes of the patients. In 7 patients some heterozygous variants were previously detected (patient no. 1–7, Table S2), the variants could be reproduced in 2 patients. For the other 5 patients, GSA could not replicate the results found from the conventional methods as the probes were not added on the array. A newly updated version of the GSA is expected to allow all these SNVs to be assessed in more detail and reveal how well they can be detected.

Next, we set out to validate the observed causal variants newly found by GSA with Sanger sequencing (*n* = 46). For 5 variants in 3 samples, Sanger sequencing could not be performed because of lack of available DNA. For the remaining 41 variants, Sanger sequencing could confirm 38 out of 41 variants (92.7%) demonstrating a high validation rate between GSA and Sanger sequencing, although some variants yielded a different nucleotide change at a slightly different position from the investigated position with the array (*n* = 8).

### CNV Analysis

**Figure 1:**
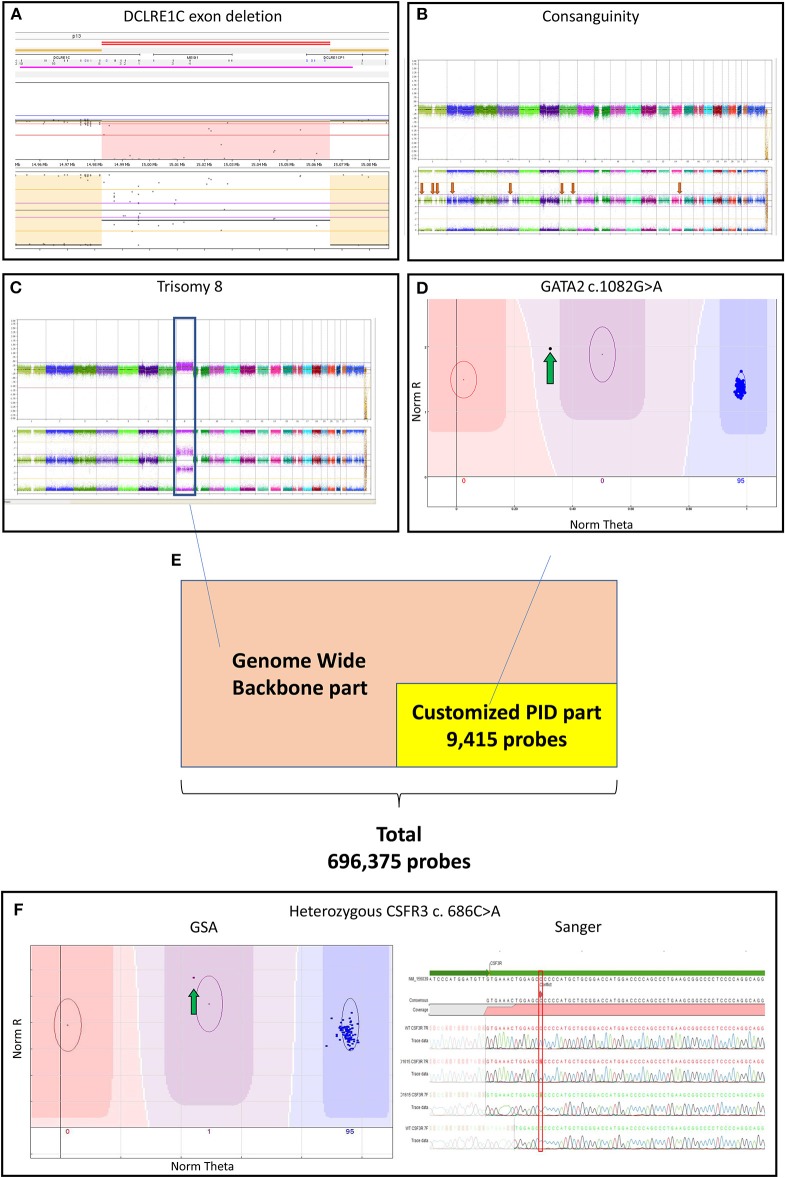
**(A)** Log-ratio plot, which shows the amount of DNA per probe (normal is two copies) and B-allele frequency plot that shows the genotype for a probe (normal is AA/AB/BB). The log-ratio plot shows a homozygous deletion depicted by the light red area in *DCLRE1C* (*Artemis*) deletion detected by CNV analysis. The call was based on 40 consecutive probes having intensity values below the threshold; **(B)** The presence of multiple regions of long-contiguous stretch of homozygosity (LCSH) in the B-allele frequency plot is suggesting consanguinity (red arrows); In a single experiment, the customized PID Global Screening Array (GSA) array identified **(C)** a trisomy 8 (blue box) seen in the log-ratio plot by amplification of DNA and seen in the B-allele frequency plot by polyploidy (AAA/AAB/ABB/BBB) by CNV calling along with **(D)** a *GATA2* mutation (green arrow) by SNV calling suggesting a secondary malignancy in the patient; **(E)** The customized GSA array comprising 9,415 PID related variants/ INDELs and a multi-ethnic genome-wide backbone on the entire array adds up to 696,375 probes; **(F)** A *CSF3R* mutation detected by SNV analysis (green arrow) was confirmed by Sanger sequencing. CNV, copy number variants*; CSF3R, colony stimulation factor 3 receptor; DCLRE1C*, DNA cross-link repair protein 1C; *GATA2, gata-binding factor 2; GSA, Global Screening Array; Sanger, Sanger sequencing;* INDELs, Insertions and Deletions; PIDs: primary immunodeficiency disorders, SNV: single nucleotide variants.

The genome-wide CNV analysis detected large inter- and intragenic regions of LCSH suggesting consanguinity in 39 PID samples (Table S1 and Figure 1B). In 12 PID patients, CNV analysis based on the array genotyping data could reveal large chromosomal aberrations and microdeletions at the gene level. Three of the previously known exon deletions that were discovered during conventional diagnostics could be reproduced, including 2 patients with a homozygous *DNA cross-link repair protein 1C (DCLRE1C)* deletion (Figure 1A) and 1 patient with a hemizygous *x-linked inhibitor of apoptosis protein (XIAP)* deletion (patient no. 3,6,27 in Table S1). One patient with an established homozygous loss of i*mmunoglobin heavy constant mu (IGHM)* could not be replicated by the GSA during CNV analysis (patient no. 34 in Table S1). Both SNV and CNV analysis detected genetic variants in one patient with a mutation in *SH2 Domain Containing 1A (SH2D1A)* (patient no. 13 in Table S1).

Interestingly, 2 PID patients without a previous genetic diagnosis were recognized as having monosomy 7, which is suspected for a hematologic malignancy rather than PID (patient no. 4,8 in Table S2). In another patient, we identified (in a single GSA experiment) both trisomy 8 (by CNV analysis) as well as a *GATA-binding factor 2 (GATA2)* variant (by SNV calling; patient no. 26 in Table S1 and Figures 1C,D), which is suggestive for a secondary hematologic malignancy in this patient.

### Sensitivity Based on Variants and Diagnostic Yield at the Patient Level

In this proof of principle study, the customized GSA array could replicate 30 out of 71 previously detected SNVs. However, 34 variants could not be investigated due to limited coverage of probes at the time of GSA design; and, 3 of the 4 previously detected CNVs could also be replicated. The sensitivity for identifying the known genetic variants (SNVs and CNVs) that the GSA can detect compared to conventional methods was 80% (33/41). Sanger sequencing could confirm 38 out of 46 newly found SNVs by GSA, although 5 variants could not be investigated due to a lack of DNA in 3 patients. The overall sensitivity including the addition of these newly found variants that underwent Sanger sequencing was 71/82 (87%) (Table S3).

**Figure 2:**
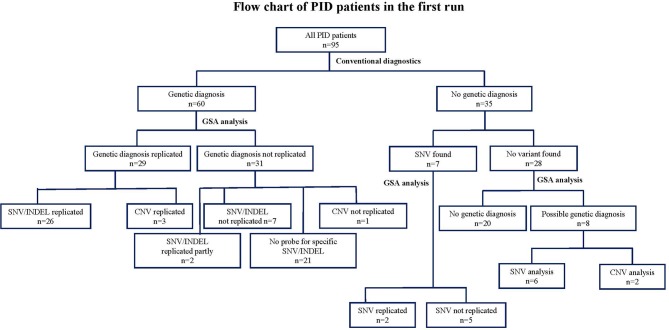
Flow chart describing the numbers of variants identified by GSA array compared to conventional methods in 95 clinically diagnosed PID patients in the first run. *CNV, copy number variants; DCLRE1C*: *GSA, Global Screening Array; PCR, Sanger sequencing; NGS, next-sequencing sequence; PID, Primary immunodeficiency disorder; SNV, single nucleotide variants*.

At the patient level, we were able to establish a genetic diagnosis using the GSA technology in 37 out of 95 PID patients (39%). These 37 patients included 29 patients in whom conventional methods had previously detected genetic variants (26 patients by SNV and 3 by CNV analysis), but also 8 newly suspected patients (6 by SNV and 2 patients suspected from leukemia as detected by CNV analysis). In 28 patients (that had established variants with conventional techniques) the variants could not be replicated by GSA due to the sparse coverage of the custom probes at the time that the array was designed and therefore these patients could not obtain a conclusive genetic diagnosis. In 20 PID patients, neither conventional nor GSA testing were able to obtain a conclusive genetic diagnosis. The detected variants leading to the diagnosis and numbers of patients in whom a diagnosis was made, comparing between conventional methods and GSA is shown in (Figure 2 and Table S3).

### Reproducibility

As indicated above, 39 PID samples could be investigated for inter-assay validation in the second run. Our results demonstrated that for 37 out of 39 patients, the genetically causal variants from the first run could be replicated in the second run (94.9%) (Tables S1, S2). Large CNVs such as large chromosomal aberrations were replicated; however, small heterozygous exon deletions did not replicate well (probably due to short primer length of the custom probes). Finally, we compared all post-QC data for the custom variants from the first run with the data generated from the second run for these 39 patients (8,883 and 8,852 variants respectively). There was overlap between 8,541 variants and we found only 13 (0.22%) differences between the genotype calls in the two runs.

### Costs

The costs for NGS in one patient including analysis in a clinical diagnostic setting is about 1,000 Euros. However, the costs depend strongly on the national economic system and the local health care infrastructure. The net price for WES without analysis and overhead varies from 350 to 600 Euros. GSA, however, can be performed for <10% of the WES price. With our effort, we performed the PID array test for the affordable price of about 40 Euros per sample. In this study, we were able to diagnose roughly 40% of patients by the array. Given a GSA cost of €40 per sample, initial costs for 100 patients is €4,000. The 60 remaining undiagnosed samples will undergo NGS or targeted gene panel sequencing assuming €1,000 per sample. Total costs for this scenario will be roughly €64,000. On the other hand, if we use directly NGS or targeted gene panel sequencing, the costs will be €100,000. For both NGS and GSA techniques, we perform Sanger sequencing for confirmation. So, by using GSA as a screening tool in PID diagnostics we can save about 36,000 Euros per 100 patients. This makes the diagnostic array an affordable promising candidate for initial screening analysis in the standard-of-care, in particular in developing countries, where genetic testing is not yet available.

## Discussion

A *high-throughput, rapid* and *inexpensive* tool is required for identifying underlying genetic defects in a clinical care setting, especially in low-income countries. SNP array technology is a powerful genomic analysis tool which has been widely used at the population level, but not for detecting rare pathogenic SNV variants.

In this study, we present a comprehensive diagnostic SNP array that is able to screen at a genome-wide level, including rare PID gene variants. This method possesses potential advantages as compared to conventional targeted gene panel NGS diagnostics. As an initial screening test, the customized PID array had a high sensitivity (87%) for capturing rare mutations and had a strong reproducibility (0.22% difference in genotype callings between two runs). Moreover, we observed a high validation accuracy compared to NGS data (0.16% difference in genotype callings). This is different from a recent previous study, which found a very low association between array and NGS data (12). However, there are important differences between the two studies. Firstly, their study used an Affymetrix/ Thermofisher product while ours used Illumina technology. SNP calling is based on different algorithms in these two platforms and thus might influence variant calling, particularly for rare variants. Secondly, we used the zCall algorithm to enhance rare variant calling, results in better genotype calling for this class of variants. A large number of newly found variants could also be reproduced by Sanger sequencing. However, there was some disagreement regarding the precise nucleotide change (n=8). Most likely, this is due to the hybridization of the probe when a variant (in the near vicinity) is present. This emphasizes the need for close investigation of the specific region with Sanger sequencing and further research on the quality of calling of individual probes.

Certainly, there is a clear trend toward implementing NGS in academic institutions for genetic diagnostics; however, there is also a trend toward array genotyping, particularly in direct-to-consumer companies. The basic cost price is 10-fold higher for NGS as compared to GSA arrays. NGS outputs a large quantity of data with concomitant much higher costs for storage and analysis. The overall price including analysis and overhead will be significantly higher for NGS than GSA. The price for NGS is expected to remain high over the next 5 years, therefore NGS will remain unaffordable in developing countries. Although NGS will still be necessary to detect novel variants, most NGS approaches for PID reported a diagnostic yield from 15 to 79% within a mixed PID population (13), with a diagnostic yield with our customized GSA of 39% falling within this range. A limitation of current NGS diagnostic approaches is that unless the DNA is sequenced with appropriate depth, NGS technology cannot accurately capture CNVs. Detection of CNVs increases the diagnostic yield by an average of 4.2% (13). Furthermore, there is no consensus about the interpretation of the variants revealed by NGS, whereas the interpretation of GSA output is simpler since the investigated variants are known beforehand.

The GSA has several clear advantages. The average turn-around-time for GSA is 1 week, therefore our economic and rapid customized PID array represents an ideal approach to broadly detecting known pathogenic variants in a primary screen, which can be followed by further NGS analysis when no genetic diagnosis can be made. Since the GSA was designed to simultaneously capture both SNVs and CNVs in a single experiment, it may also indicate secondary malignancies in at-risk PID patients. This is illustrated in our study by the co-occurrence of a *GATA2* variant and a CNV aberration of chromosome 8, which is indicative for acute myeloblastic leukemia (14). Due to the complexity and variety of clinical presentations, hematological malignancies can be misdiagnosed as a PID. We also detected excessive LCSH in some of our patients, which is clearly indicating consanguinity. Such LCSH areas are likely to reflect the origin of recessive diseases, such as imprinting disorders, triploidy and duplications in which the pathogenic mutations are located (4). Although not observed in this study, 22q11 microdeletion syndrome (4) and IL-25 hyperdiploidy can be discovered by CNV analysis (15). SNVs related to inflammatory and autoimmune complications in common variable immunodeficiency (CVID) were reported (16). SNV analysis is also capable of detecting a Uniparental Disomy as shown recently in a patient with *lipopolysaccharide (LPS)-responsive and beige-like anchor protein* (*LRBA*) Deficiency (17).

Finally, enriched GSA with hotspot mutations enable the screening for other immune-mediated diseases such as Blau syndrome (NOD2), mastocytosis (KIT), and as cancer driver mutations in proto-oncogenes such as *v-raf murine sarcoma viral oncogene homolog B1 (BRAF)* or tumor suppressor genes such as *tumor protein* 53 *(TP53)*. Therefore, a similar approach of an enriched array with known pathogenic variants may be a promising screening tool for other genetic diseases. Although it is unlikely that the array will work better for one disease than another, trials should be performed to conclusively demonstrate the utility of arrays for detecting rare disease-causing genetic variants in a range of genetic disorders.

Despite its advantages, this customized array has some limitations. Firstly, it is unable to detect novel SNV, i.e., those that have not yet been reported in literature and/or databases, it can only detect variants that are included on the array. However, since GSA is scalable, it is possible to increase the number of variants in additional genes. The current version of our customized array contained only selected variants of 277 PID-causing genes derived from the IUIS 2015 classification (as published at the time the array was designed). The total number of known PID genes has now vastly increased, with 420 inborn errors of immunity as described in the updated IUIS 2019 classification (3). By updating the GSA with newly found variants, the diagnostic yield is expected to improve significantly. This warrants for further assessment of the GSA as a primary screening tool. Although we cannot test every probe by validation with known carriers and cannot guarantee for possible probe malfunction, we expect no technical reasons (given the high accuracy rate compared with NGS and the high validation rate compared with Sanger sequencing) why the sensitivity and diagnostic yield should not increase accordingly. Indeed, it should be possible to create a user-friendly, automated platform that analyzes data efficiently, thus benefiting medical decision making in patients with PIDs.

In conclusion, this robust customized array is a promising first-line rapid screening tool for PID and probably other genetic diseases at an affordable cost. This method provides new perspectives for genetic testing in developing countries where PIDs are currently under-diagnosed.

## Data Availability Statement

The datasets for this article are not publicly available because of the legal and ethical restrictions, as this will compromise the anonymity of the participants in the study, according to the GDPR related restrictions.

## Ethics Statement

The studies involving human participants were reviewed and approved by MREC Erasmus Medical Center Rotterdam. Written informed consent to participate in this study was provided by the participants' legal guardian/next of kin.

## Author Contributions

NS and RW contributed equally to this work through writing the manuscript, data collection, and data analysis. LB, JM, and AU performed data analysis and wrote the manuscript. LX, BB, MB, and WD wrote the manuscript and supported the clinical data. SS, JG, IM, VD, KS, PC, and JD wrote the manuscript. AL and KH performed Sanger sequencing and wrote the manuscript. PS and PH wrote the manuscript and took overall control of this work. The SEAPID Consortium Members were involved in the conceptual design of the study and writing the manuscript.

### Conflict of Interest

The authors declare that the research was conducted in the absence of any commercial or financial relationships that could be construed as a potential conflict of interest.

## Abbreviations

BRAF: v-raf murine sarcoma viral oncogene homolog B1
BTK: bruton tyrosine kinase
CNV: copy number variants
CSF3R: colony stimulation factor 3 receptor
CVID: common variable immunodeficiency
CXCR-4: C-X-C chemokine receptor type 4
DCLRE1C: DNA cross-link repair protein 1C
GATA2: gata-binding factor 2
GSA: global screening array
GWAS: genome-wide association study
IKBKG: inhibitor of nuclear factor kappa B kinase subunit gamma
INDELs: Insertions and Deletions
IUIS: the International Union of Immunological Studies
LCSH: long-contiguous stretch of homozygosity
LRBA: lipopolysaccharide (LPS)-responsive and beige-like anchor protein
NGS: next-generation sequencing
PIDs: primary immunodeficiency disorders
SNP: single nucleotide polymorphism
SNV: single nucleotide variants
STAT1: signal transducer and activator of transcription 1
TNFRSF13B: tumor necrosis factor receptor superfamily member 13B
TP53: tumor protein 53
VPS45: vacuolar protein sorting 45 homolog
WAS: wiskott–aldrich syndrome
WGS: whole-genome sequencing
XIAP: x-linked inhibitor of apoptosis protein.
