# Supplementary material for: Rapid Low-Cost Microarray-Based Genotyping for Genetic Screening in Primary Immunodeficiency
Source: Front Immunol. 2020 Apr 15;11:614. doi: 10.3389/fimmu.2020.00614 (PMC7179678; doi:10.3389/fimmu.2020.00614)
Supplement: Supplementary file 3 [file Table_3.pdf]

Table E3: **Comparison of causal genetic variant detection between conventional methods and the customized GSA**

**Item**	**Conventional Methods(Numbers)**		**GSA technology (Numbers)**
**Method**	**Previously detected variants**	**Known variants that could be investigated by GSA**	**Replicated variants by GSA**
SNV	71 *	37	30
CNV	4	4	3
**Method**	**Newly found variants replicated by Sanger sequencing**	**Newly found variants that could be investigated by Sanger sequencing**	**Newly found variants by GSA**
SNV	38	41	46**
CNV	none	none	2

* 34 out of 71 variants did not include when the GSA was designed.

** 5 out of 46 variants could not be investigated (lack of DNA).

GSA: Global Screening Array, CNV: copy number variants, SNV: single nucleotide variants
